# Functionalized Biochars Derived from Tomato Plant Residues (*Solanum lycopersicum* L.) and Shea Nut Shells (*Vitellaria paradoxa*) for Phosphorus Sorption: From Model Solutions to Real Wastewater Matrices

**DOI:** 10.3390/molecules31183277

**Published:** 2026-09-16

**Authors:** Maja Owczarek, Hanna Siwek, Kacper Świechowski

**Affiliations:** 1 Department of Bioengineering, West Pomeranian University of Technology in Szczecin, Słowackiego 17, 71-434 Szczecin, Poland; hanna.siwek@zut.edu.pl; 2 Department of Applied Bioeconomy, Wrocław University of Environmental and Life Sciences, Chełmońskiego 37a, 51-630 Wrocław, Poland; kacper.swiechowski@upwr.edu.pl

**Keywords:** biochar, phosphate sorption, magnetic modification, diatomite incorporation, wastewater treatment, agricultural waste valorization

## Abstract

Waste biomass can serve as a feedstock to produce functionalized biochars (BCs) for phosphate removal. However, their sorption performance depends on feedstock properties, pyrolysis conditions, and modification strategy. This study compared BCs produced from tomato plant residues (*Solanum lycopersicum* L.; T) and shea nut shells (*Vitellaria paradoxa*; VP), pyrolyzed at 400 and 500 °C and modified with iron (Fe) or diatomite (DT). Their physicochemical and structural properties and phosphate sorption capacity were evaluated in a model solution and selected real wastewater matrices. Exploratory variance decomposition showed that modification type represented the largest proportion of variability in physicochemical and structural properties of 12 BC variants (59.75%), whereas feedstock type represented 84.84% of the observed variation in phosphate sorption across the 8 variants. The effect of modification was feedstock-dependent: DT was particularly beneficial for T-derived BCs, whereas Fe modification was more beneficial for VP-derived BCs. Selected magnetic BCs also removed phosphate from real wastewater matrices, although removal efficiency differed between matrices. Overall, phosphate sorption capacity could not be predicted from modification type or pyrolysis temperature alone, indicating that feedstock characteristics should be considered when selecting functionalization strategies.

## 1. Introduction

The uncontrolled discharge of phosphorus (P) from agricultural and municipal wastewater contributes to the eutrophication of receiving waters and remains a major environmental concern. Approximately 48% of wastewater generated globally is estimated to be discharged untreated, while global wastewater production reaches 359.4 · 10^9^ m^3^·yr^−1^ [1]. P is essential for food production, while its primary supply relies largely on non-renewable phosphate rock resources, with global demand projected to increase by 2.5% annually [2]. Recovery of P from wastewater has been estimated to potentially meet 15–20% of global fertilizer P demand, thereby reducing reliance on primary phosphate resources and supporting more sustainable nutrient management. Consequently, the recovery of P and other nutrients from waste streams represents an important component of sustainable resource management and the circular economy. Sorption-based approaches are particularly attractive in this context because they can combine efficient phosphate removal with the potential for subsequent valorization of the P-loaded sorbent. Depending on the sorbent properties and the form and strength of P binding, spent sorbents may potentially be used as nutrient carriers with slow-release characteristics or subjected to desorption and regeneration. In this context, biochar (BC) represents a promising sorbent produced through the thermochemical conversion of biomass under oxygen-limited conditions. BC is a carbon-rich solid material whose production can require substantially less energy (1.1–16 MJ·kg^−1^) than that of activated carbon (44–170 MJ·kg^−1^) [3]. The ability of BC to retain and subsequently release nutrients is influenced by its specific surface area, porosity, oxygen-containing functional groups, and mineral composition. These properties are strongly affected by feedstock characteristics, pyrolysis conditions, and post-production modification.

Pyrolysis temperature is one of the key process parameters affecting BC properties. In general, increasing pyrolysis temperature promotes devolatilization and carbonization, often resulting in increased aromaticity and structural stability and, in many cases, a greater specific surface area. At the same time, higher temperatures reduce the abundance of oxygen-containing functional groups [4,5], alter the relative contribution of the mineral fraction [6], and typically decrease BC yield [7]. At lower pyrolysis temperatures, BCs generally exhibit lower specific surface areas but retain a greater abundance of oxygen-containing functional groups, which may provide chemically reactive sites contributing to sorption processes [4].

Feedstock type is another major factor influencing the physicochemical and structural properties of BC. The chemical and mineral composition of the precursor biomass affects the resulting carbon matrix, pore development, surface chemistry, and distribution of functional groups [8]. The use of waste-derived biomass, including agricultural and agro-industrial residues, as a feedstock for BC production additionally provides a pathway for the valorization of residual biomass streams [4].

Because pristine BCs may exhibit limited affinity for phosphate, various physical, chemical, and biological modification strategies have been investigated to enhance their sorption performance. These include physical treatments such as steam activation and milling [9], biological modification through microbial immobilization [10], the formation of composites with zeolites or clay minerals [7,11], and acid or alkali treatments that can alter pore accessibility and surface functional groups [9]. Metal-based modification (impregnation) using magnesium (Mg), calcium (Ca), aluminium (Al), or iron (Fe) is particularly relevant to phosphate sorption because it introduces reactive metal-containing sites capable of interacting with phosphate species [12]. Fe modification can additionally impart magnetic properties to BC, facilitating its separation from the aqueous phase after treatment [11,13].

Despite extensive research on phosphate sorption by BCs, DT-containing BC composites have received comparatively limited attention, particularly in studies directly comparing materials derived from chemically distinct waste biomass feedstocks produced and modified under identical conditions. Therefore, this study investigated the effects of waste feedstock type, pyrolysis temperature (400 and 500 °C) and modification strategy (DT-containing composite or Fe-based magnetic modification) on the physicochemical and structural properties of BC and its phosphate sorption performance under model conditions. Two contrasting waste biomass feedstocks were investigated: tomato plant residues (*Solanum lycopersicum* L.; T) and shea nut shells (*Vitellaria paradoxa*; VP). Selected magnetic BCs were additionally evaluated in real wastewater matrices to assess their sorption behavior under more chemically complex and matrix-dependent conditions.

## 2. Results

### 2.1. Physicochemical and Structural Properties of Biochar

The physicochemical composition of the investigated feedstocks and the resulting BCs indicated that the effects of pyrolysis and modification were strongly dependent on the initial feedstock. Total C content and mineral composition differed between BCs derived from the two biomass types. The T and VP feedstocks (Supplementary Materials, Table S1) had comparable C and N contents but differed significantly in their mineral composition. T biomass contained significantly higher amounts of ash, Mg, and Ca, whereas VP biomass was characterized by higher P, K, and Fe contents.

The elemental composition of the resulting BCs showed higher carbon (C) contents in VP-derived BCs than in T-derived BCs (Table 1). At both pyrolysis temperatures, VP400 and VP500 contained more than 50% C, whereas the C content of T-derived BC decreased from 47.65% in T400 to 24.27% in T500. This decrease was accompanied by a marked increase in the concentrations of several mineral elements, indicating a greater relative contribution of the inorganic fraction at the higher pyrolysis temperature. For all BC variants, increasing the pyrolysis temperature was accompanied by a decrease in N content. No consistent trend was observed for S, indicating that its concentration varied depending on both the feedstock and modification treatment.

Modification altered the elemental composition of the BCs. DT-containing composites generally contained lower concentrations of C and N than their corresponding unmodified BCs, which is consistent with a lower proportion of the carbonaceous component in these materials. In most cases, the DT-containing composites also showed lower K, Ca, and Mg concentrations. Accordingly, these materials exhibited a substantially different bulk elemental composition from the corresponding unmodified BCs. Fe modification resulted in a pronounced enrichment of the samples with Fe, consistent with the incorporation of an Fe-containing phase. This enrichment was accompanied by lower concentrations of several other elements, which may reflect both dilution by the introduced Fe-containing phase and partial removal of soluble constituents during the modification procedure.

The specific surface area (S_BET_) of the investigated BCs ranged from 1.21 to 130.12 m^2^·g^−1^ (Table 2). The highest S_BET_ values were observed for Fe-modified BCs. Depending on feedstock and pyrolysis temperature, Fe-modified samples exhibited S_BET_ values ranging from 51.71 to 130.12 m^2^·g^−1^, compared with 2.37–9.92 m^2^·g^−1^ for their corresponding unmodified BCs. This corresponded to approximately 11-, 13-, 19-, and 42-fold increases for VP400, T400, T500, and VP500, respectively. A similar trend was observed for the DFT-derived surface area (S_DFT_) and cumulative pore volume (V_DFT_). Thus, Fe modification was consistently associated with increased N_2_-accessible surface area and pore volume. In contrast, the incorporation of DT decreased S_BET_ by 37–70% relative to the corresponding unmodified BCs. Increasing the pyrolysis temperature from 400 to 500 °C was associated with lower S_BET_ values in both unmodified and DT-containing BCs.

**Figure 1:**
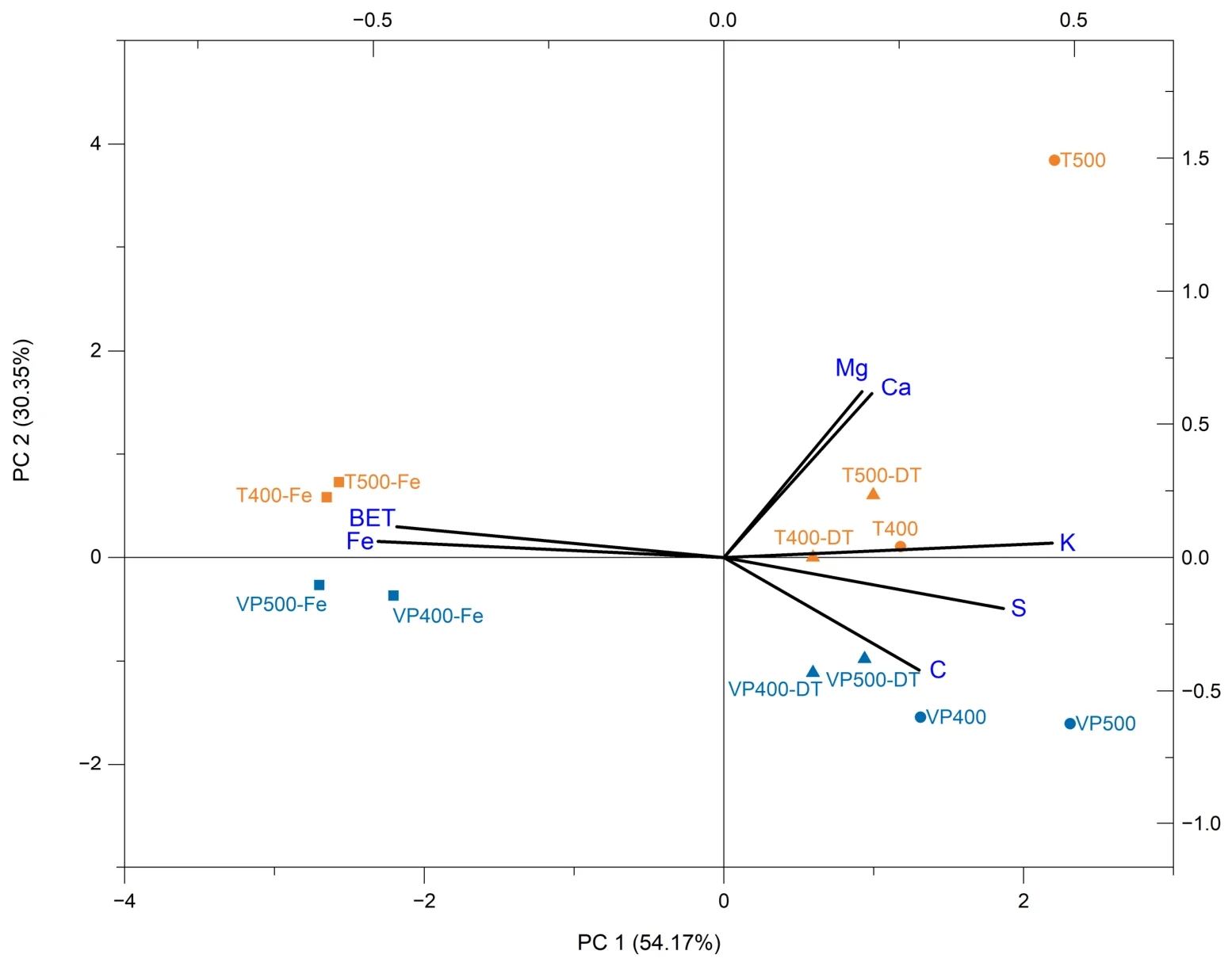
Principal component analysis (PCA) of the physicochemical and structural properties of unmodified and modified biochars (BCs). Orange and blue symbols represent tomato-derived (T) and shea nut shell-derived (VP) BCs, respectively; circles, triangles, and squares denote unmodified, DT-containing, and Fe-modified BCs, respectively. C—carbon; S—sulfur; Ca—calcium; Mg—magnesium; K—potassium; Fe—iron; and BET—BET specific surface area.

Exploratory variance decomposition of the BC properties (*n* = 12) showed that modification type represented 59.75% of the observed variability, whereas feedstock type and pyrolysis temperature represented 16.4% and 5.4%, respectively (Supplementary Materials, Table S2). This pattern was also reflected in the principal component analysis (PCA) of the same dataset (Figure 1). The first two principal components explained 84.52% of the total variance, with PC1 and PC2 accounting for 54.17% and 30.35%, respectively.

Fe-modified BCs were positioned along the negative PC1 axis, consistent with the high negative loadings of Fe content, S_BET_, and cumulative pore volume on this component. These samples clustered together irrespective of feedstock type and pyrolysis temperature. PC2 primarily differentiated samples with higher Ca and Mg contents from those associated with higher C content. T500 occupied the most positive position along PC2, whereas unmodified VP-derived BCs were oriented toward the C and S loading vectors. In contrast to Fe modification, the incorporation of DT did not result in a distinct clustering pattern.

#### Fourier Transform Infrared Spectroscopy (FT-IR)

**Figure 2:**
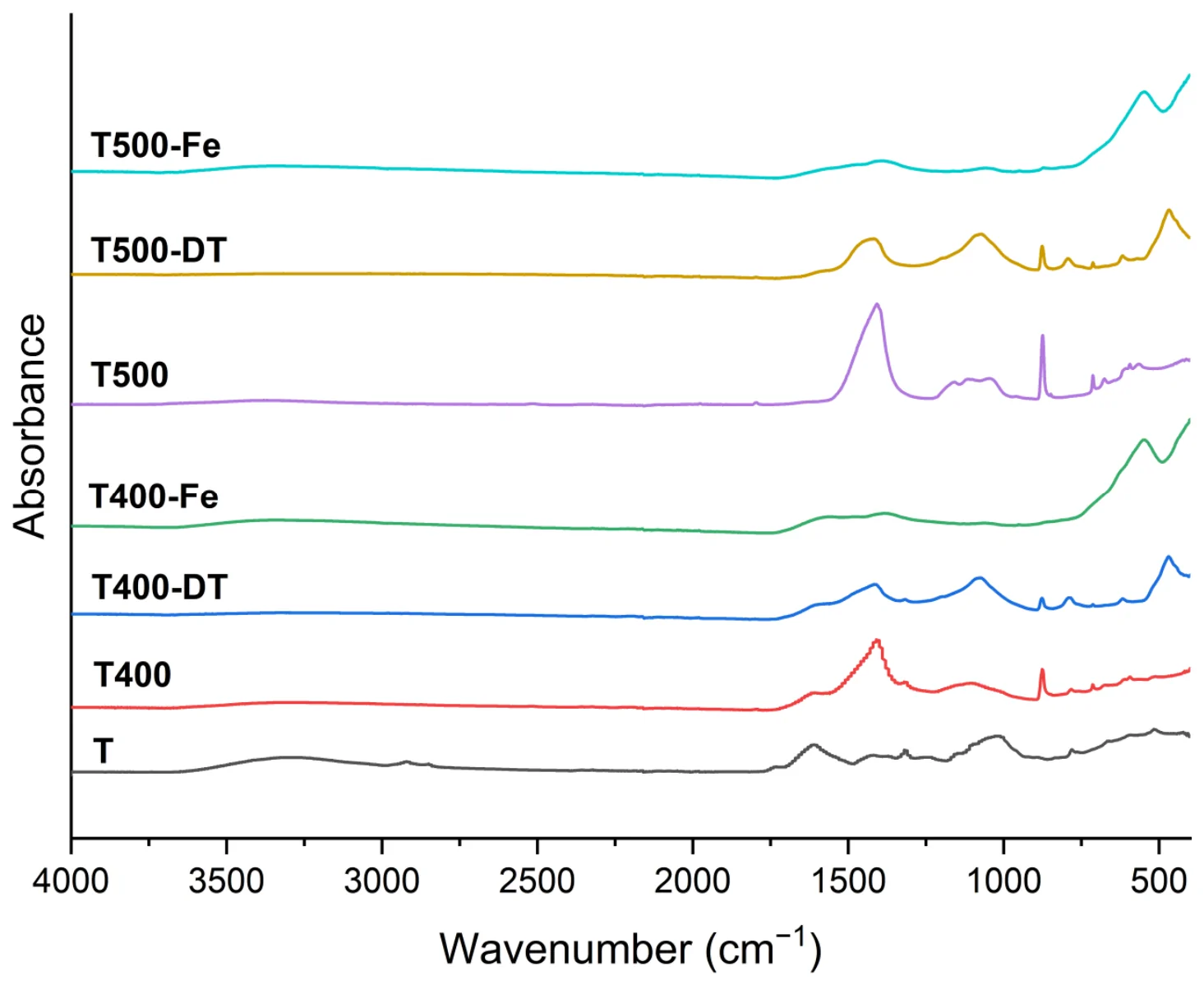
FT-IR spectra of tomato plant residues (T) and the corresponding unmodified and modified BCs produced at 400 and 500 °C.

**Figure 3:**
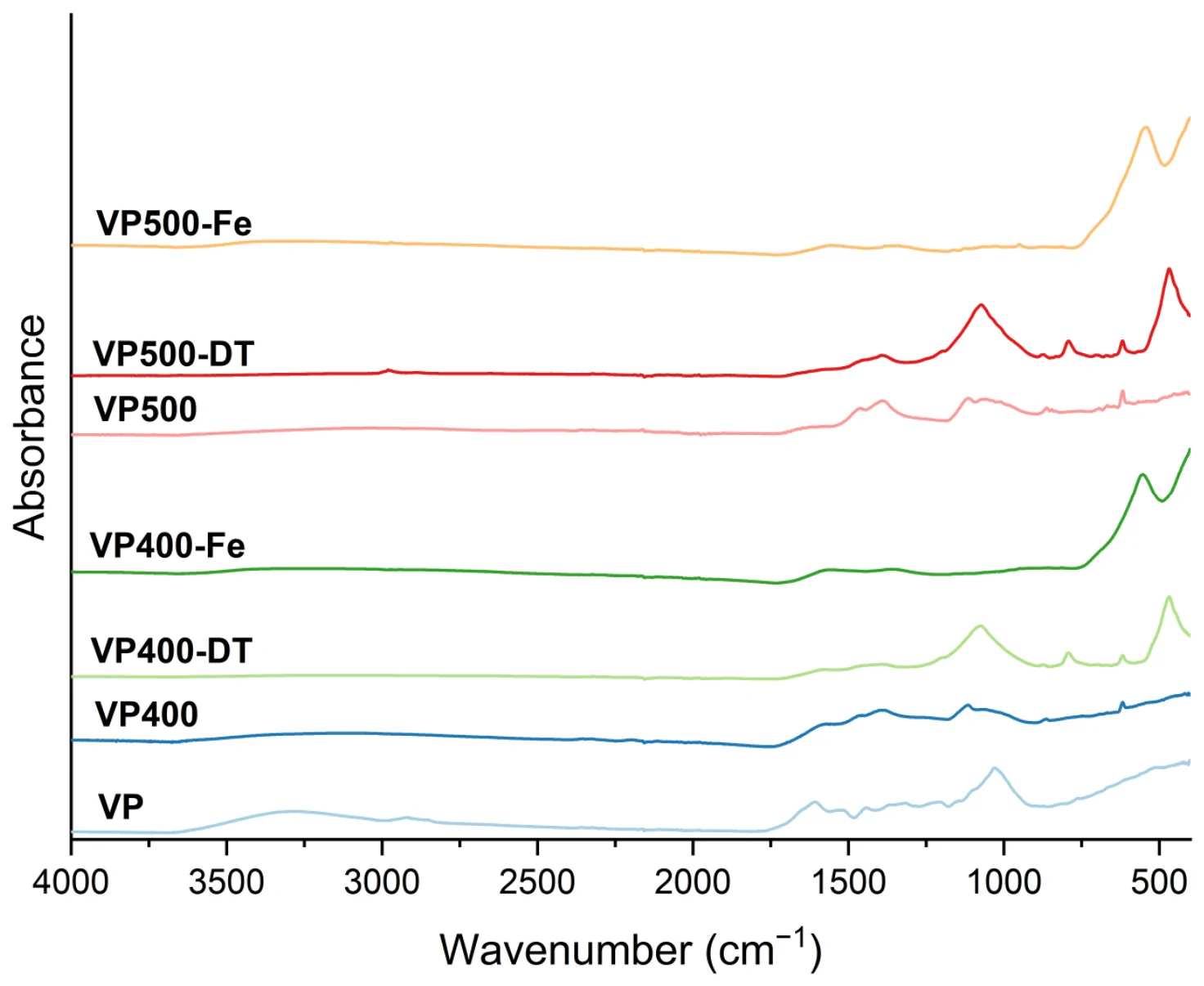
FT-IR spectra of shea nut shells (VP) and the corresponding unmodified and modified BCs produced at 400 and 500 °C.

The FT-IR spectra of the feedstocks and the corresponding unmodified and modified BCs produced at 400 and 500 °C are shown in Figure 2 and Figure 3. The FT-IR spectrum of DT used for the preparation of the BC-DT composites is provided in the Supplementary Materials (Figure S1).

A broad band at approximately 3300 cm^−1^, attributed to O-H stretching vibrations, was observed in most T-derived samples and selected VP-derived samples. Bands in the 3000–2800 cm^−1^ region, associated with aliphatic C-H stretching vibrations, were also observed in T-derived and selected VP-derived samples [14]. Bands in the approximately 1600–1500 cm^−1^ region were attributed mainly to aromatic C=C skeletal vibrations [15]. The bands observed at approximately 1400–1380 cm^−1^, particularly in the T series, may include contributions from both aliphatic C-H deformation and carbonate-related vibrations. In the feedstocks and unmodified BCs, bands within the 1220–1020 cm^−1^ region were primarily associated with C-O and C-O-C stretching vibrations. A band near 870 cm^−1^ may be associated with out-of-plane aromatic C-H bending vibrations [14]. However, a contribution from carbonate species cannot be excluded, particularly in the mineral-rich T-derived BCs.

The incorporation of DT resulted in characteristic spectral features at approximately 1075 cm^−1^, 790 cm^−1^ and 470 cm^−1^. The band at approximately 1075 cm^−1^ was assigned predominantly to asymmetric Si-O-Si stretching, whereas the bands near 790 cm^−1^ and 470 cm^−1^ were associated with Si-O-Si or Si-O-Al vibrations, consistent with the presence of siliceous and aluminosilicate components derived from DT [16]. The Si-O-related spectral features were more pronounced in BC-DT composites produced at 500 °C. In Fe-modified BCs, a band at approximately 550–540 cm^−1^ was attributed to Fe-O vibrations, consistent with the presence of Fe-containing oxide species [17].

### 2.2. Phosphate Sorption Kinetics

The kinetic parameters obtained by fitting the pseudo-second-order (PSO) model to the phosphate sorption data are presented in Table 3.

**Figure 4:**
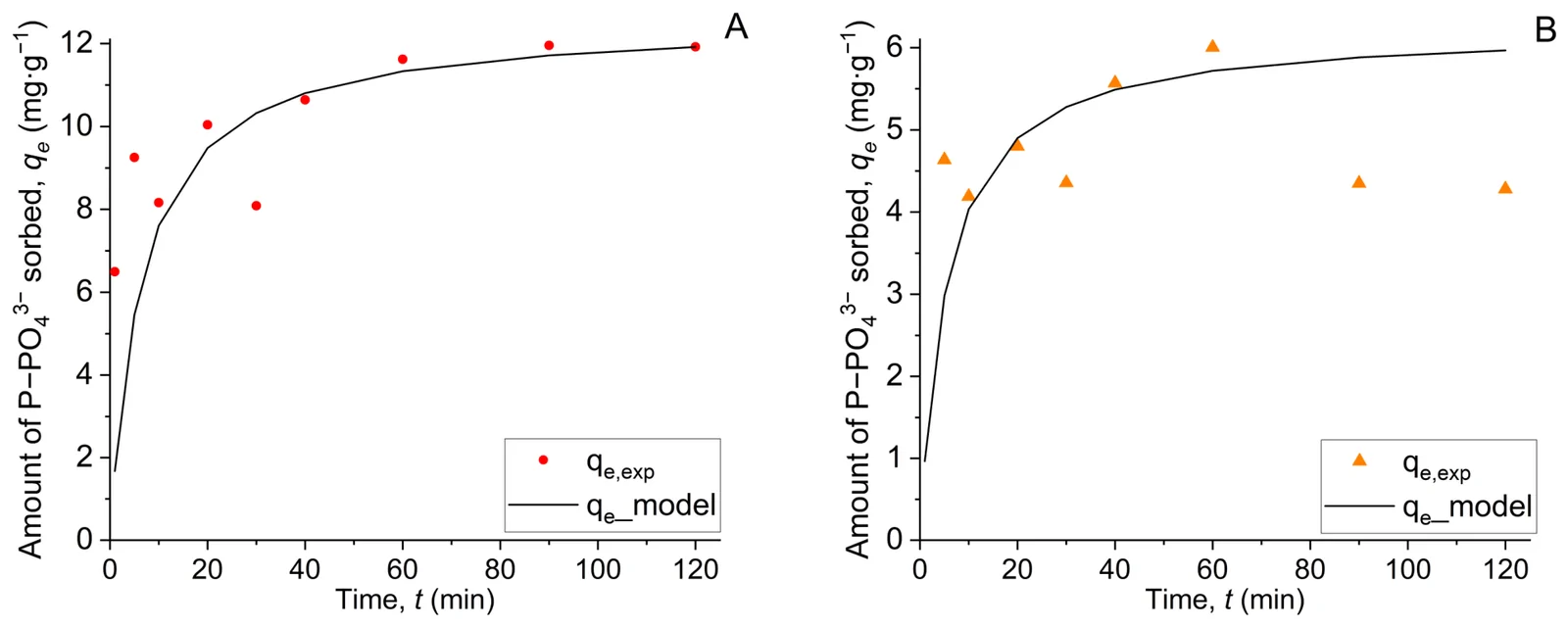
Representative phosphate sorption kinetic profiles for (**A**) tomato-derived T500-Fe and (**B**) shea nut shell-derived VP500-Fe magnetic biochars. Symbols represent experimental data, while lines indicate pseudo-second-order model fits.

The PSO model described the phosphate sorption kinetics with varying goodness of fit among the investigated BCs. For samples for which the model parameters could be reliably determined, *R*^2^ values ranged from 0.908 to 0.988, indicating a relatively good statistical fit of the PSO model to the experimental data. Lower model stability was observed primarily for systems exhibiting only small changes in the amount of phosphate sorbed at time (*q_t_*) during the experiment, such as VP500-DT. Unmodified BCs generally exhibited relatively low phosphate uptake. For samples designated as n.d., the magnitude of the sorption response relative to experimental variability was insufficient to obtain stable and meaningful PSO parameter estimates. Selected phosphate sorption kinetic profiles are shown in Figure 4.

The highest *q_e,exp_* value was obtained for T500-DT (15.75 mg·g^−1^), followed by the Fe-modified T-derived BCs, T500-Fe (11.92 mg·g^−1^) and T400-Fe (11.31 mg·g^−1^). Within the VP series, the highest *q_e,exp_* values were observed for the Fe-modified BCs, VP400-Fe (4.65 mg·g^−1^) and VP500-Fe (4.28 mg·g^−1^). For Fe-modified BCs, increasing the pyrolysis temperature from 400 to 500 °C was associated with only minor differences in *q_e,exp_* within each feedstock series. At the same time, T-derived BC-DT composites exhibited relatively high *q_e,exp_* values despite their low S_BET_ values, whereas VP-derived BC-DT composites showed substantially lower phosphate uptake.

The parameter *h* reflected differences in the model-predicted initial sorption rate. Despite their similar *q_e,exp_* values, VP400-Fe exhibited an almost twofold higher *h* value than VP500-Fe (2.177 vs. 1.145 mg·g^−1^·min^−1^). Interpretation of *k*_2_ should be considered together with *q_e,calc_* and the goodness of model fit (*R*^2^), particularly for systems characterized by low sorption. The kinetic profiles shown in Figure 4 indicated that the most pronounced changes in phosphate uptake occurred within the first 40 min of contact, followed by slower changes in *q_t_*.

### 2.3. Phosphate Sorption Isotherms

The Langmuir and Freundlich isotherm parameters obtained for phosphate sorption onto the modified BCs are presented in Table 4.

**Figure 5:**
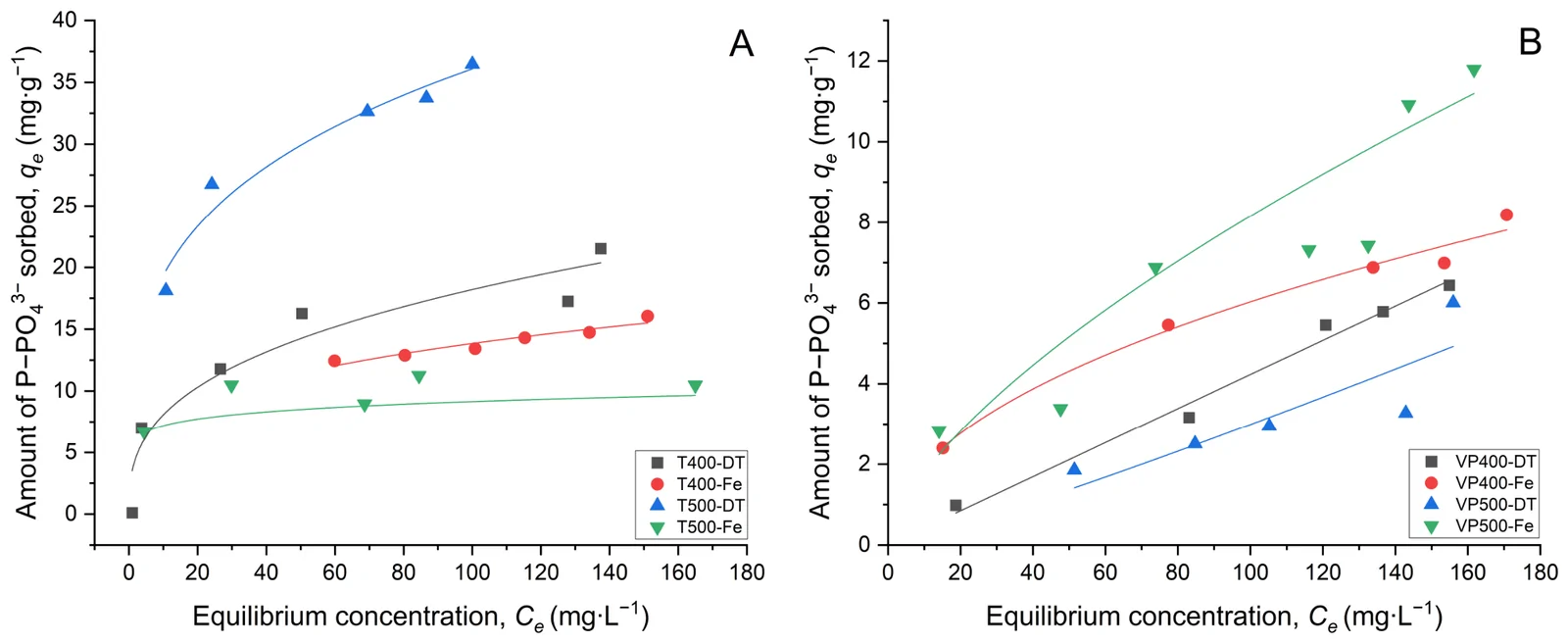
Phosphate sorption isotherms for modified biochars derived from (**A**) tomato plant residues (T) and (**B**) shea nut shells (VP). Symbols represent experimental data, while lines indicate Freundlich model fits.

Comparison of the Langmuir and Freundlich models showed that the Freundlich equation provided a better description of phosphate sorption, as indicated by higher *R*^2^ values for most of the investigated BCs (Table 4). This pattern is consistent with sorption occurring on energetically heterogeneous surfaces. An exception was T500-DT, for which the Langmuir model showed a better fit (0.983) than the Freundlich model (0.960). For both VP400-DT and VP500-DT, the Langmuir model resulted in very high *q_max_* values accompanied by very low *K_L_* values, which, together with the absence of a clear sorption plateau, indicate that *q_max_* estimates were strongly dependent on extrapolation beyond the experimentally observed range. The Freundlich model provided satisfactory fits for most of the investigated systems, with *R*^2^ values ranging from 0.912 to 0.986, except for T500-Fe (0.640) and VP500-DT (0.722). For T500-Fe and VP500-DT, both models provided poor fits, indicating that neither model adequately described the experimental sorption behavior. According to the Giles classification, the isotherms of T-derived BCs and Fe-modified VP-derived BCs (Figure 5) exhibited predominantly L-type behavior, indicating a progressive decrease in the availability of energetically favorable sorption sites with increasing phosphate concentration. In contrast, the DT-containing VP-derived composites exhibited nearly linear isotherms consistent with C-type behavior, suggesting an approximately constant partitioning of phosphate between the aqueous phase and the sorbent over the investigated concentration range [18].

Among the T-derived BCs with satisfactory Freundlich fits, T500-DT exhibited the lowest 1/*n* value (0.271), indicating the strongest deviation from linear sorption behavior. The same sample also showed the highest *K_F_* value (10.36), whereas T400-DT exhibited a lower *K_F_* value (3.569). For both modification strategies within the T series, BCs produced at 500 °C exhibited higher *K_F_* values than the corresponding materials produced at 400 °C. In the VP series, *K_F_* values were generally lower and 1/*n* values higher than those observed for the T-derived BCs. The DT-containing VP-derived composites exhibited particularly low *K_F_* values (0.042 and 0.017) and 1/*n* values close to 1, consistent with their nearly linear isotherm profiles. For VP-derived BCs, increasing the pyrolysis temperature from 400 to 500 °C was associated with lower *K_F_* values for both Fe- and DT-containing materials.

**Figure 6:**
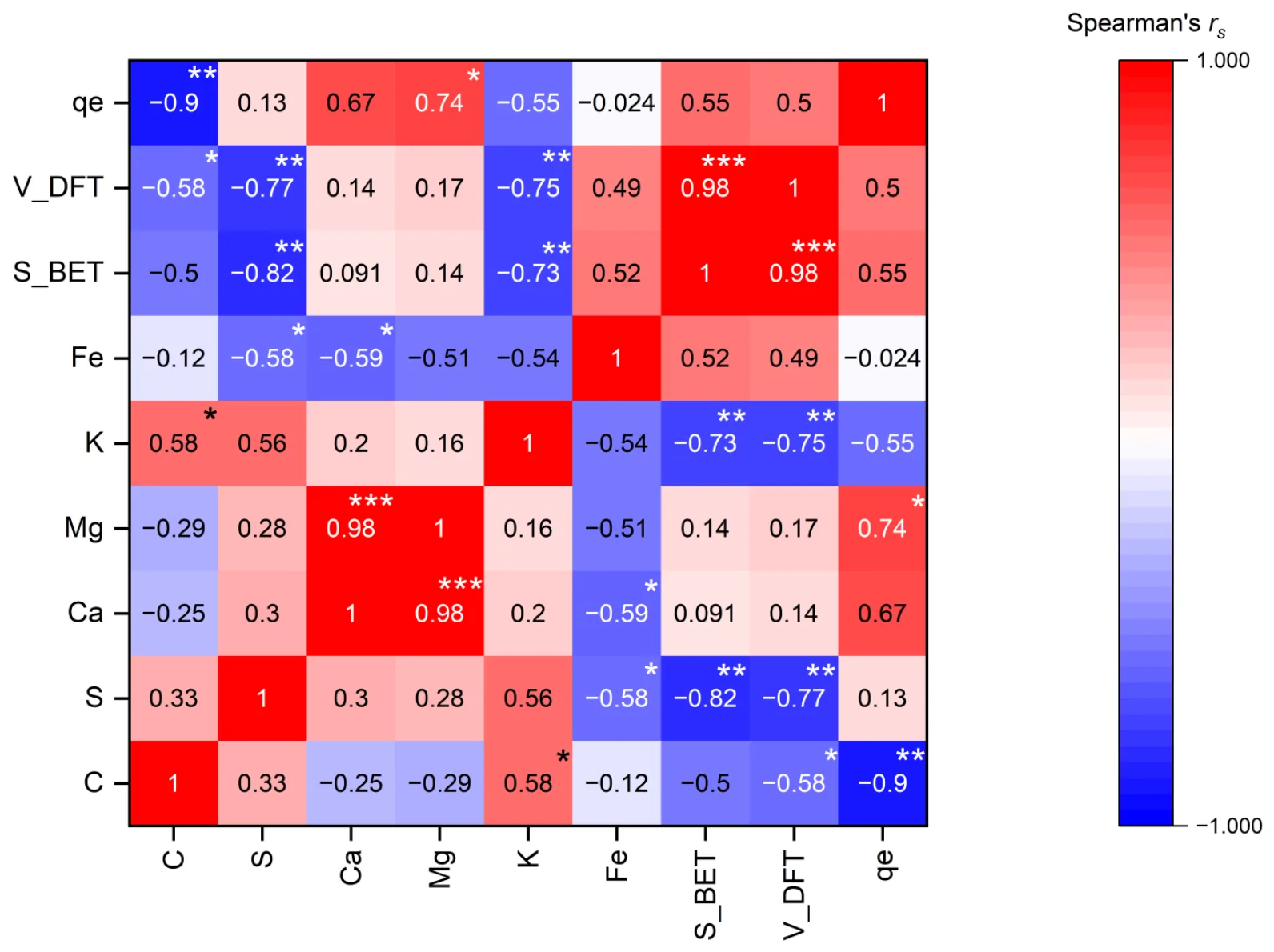
Heatmap of Spearman’s rank correlation coefficients (*r_s_*) between selected physicochemical and structural properties of the biochars (*n* = 8) and experimentally determined phosphate sorption amount. Asterisks indicate statistical significance based on two-tailed tests: *p* ≤ 0.05 (*), *p* ≤ 0.01 (**), and *p* ≤ 0.001 (***). C—carbon; S—sulfur; Ca—calcium; Mg—magnesium; K—potassium; Fe—iron; S_BET_—specific surface area; V_DFT_—cumulative pore volume; q_e_—experimentally determined sorption amount at 120 min.

Exploratory variance decomposition based on 8 BC variants (*n* = 8) showed that feedstock type represented 84.84% of the observed variability in *q_e_*, whereas pyrolysis temperature and modification type represented 1.49% and 0.69%, respectively (Supplementary Materials, Table S3). Spearman’s rank correlations between the selected BC properties and the experimentally determined sorption amount are presented as a correlation heatmap in Figure 6.

Spearman’s rank correlation analysis showed several associations among the investigated BC properties. S_BET_ was strongly positively correlated with cumulative pore volume (*r_s_* = 0.98), and a similarly strong positive correlation was observed between Ca and Mg contents (*r_s_* = 0.98). Phosphate sorption capacity (*q_e_*) was positively correlated with S_BET_ (*r_s_* = 0.55), pore volume (*r_s_* = 0.50), Ca content (*r_s_* = 0.67), and Mg content (*r_s_* = 0.74). The correlation between *q_e_* and Fe content was close to zero, indicating no apparent monotonic association between these variables. Despite the positive correlation between S_BET_ and *q_e_*, BCs with the highest S_BET_ values did not consistently exhibit the highest phosphate uptake.

### 2.4. Phosphate Removal from Real Wastewater Matrices

The physicochemical characteristics of the FL and SL matrices are summarized in Table 5. FL was characterized by substantially higher initial phosphate concentration, electrical conductivity, sulfate concentration, and total alkalinity, whereas SL exhibited higher pH and total hardness. Overall, the two wastewater matrices differed markedly not only in phosphate loading but also in their physicochemical characteristics and buffering capacity.

**Figure 7:**
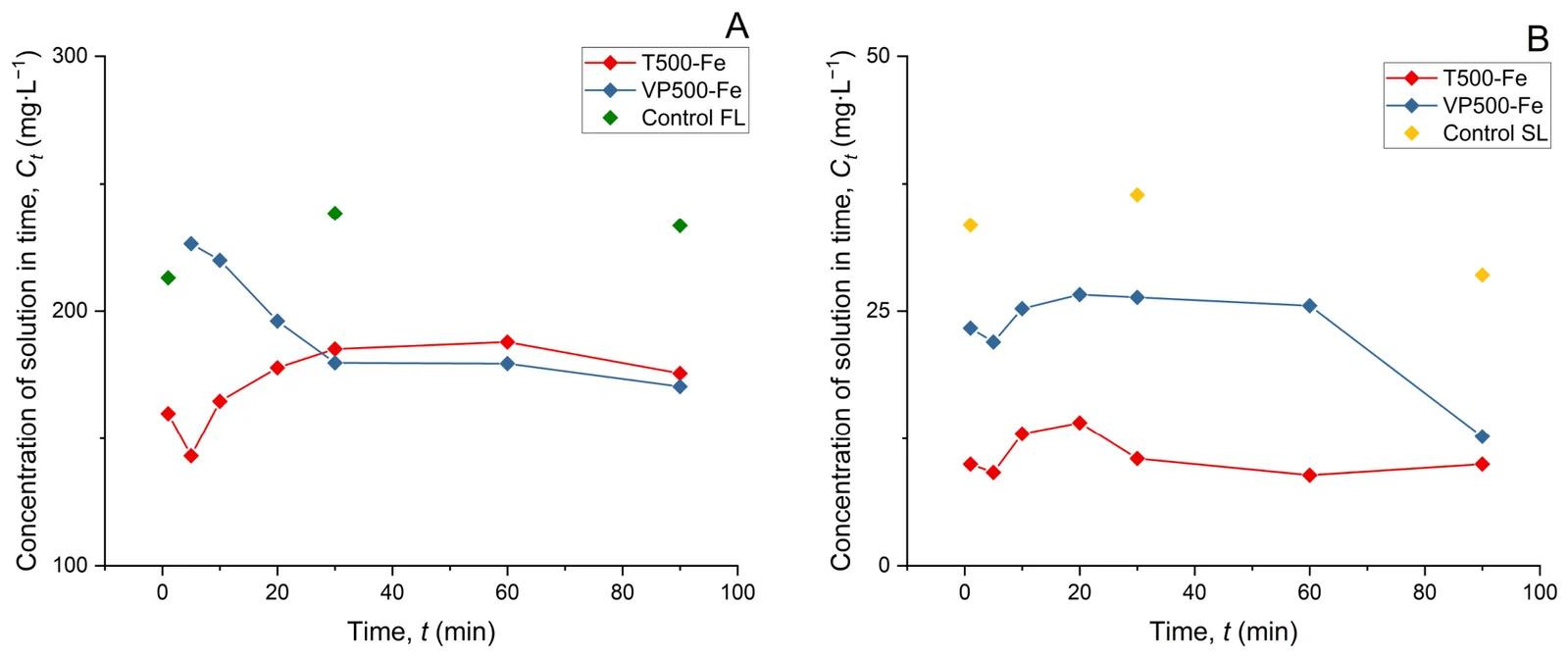
Changes in dissolved P−PO_4_^3−^ concentrations during batch contact of (**A**) feed-production leachate (FL) and (**B**) sludge leachate (SL) with the magnetic biochars T500-Fe and VP500-Fe. Control samples consisted of the corresponding wastewater matrix without biochar.

Changes in dissolved phosphate concentrations in selected real wastewater matrices during contact with magnetic BCs are shown in Figure 7.

In the FL matrix, both T500-Fe and VP500-Fe reduced phosphate concentrations relative to the corresponding control without sorbent addition (Figure 7A). After 90 min of contact with T500-Fe, the phosphate concentration was approximately 25% lower than in the control. A similar reduction was observed for VP500-Fe, reaching approximately 27%. Phosphate concentrations showed a non-monotonic temporal pattern in both FL treatments.

A different temporal pattern was observed in the SL matrix (Figure 7B). For T500-Fe, phosphate removal reached approximately 65% during the initial stage of contact, followed by only minor changes at subsequent sampling times. VP500-Fe exhibited a lower initial removal efficiency, whereas a more pronounced decrease in phosphate concentration occurred at later contact times. After 90 min, phosphate removal by VP500-Fe reached approximately 55%, approaching that observed for T500-Fe. Such distinct differences between the temporal profiles of T500-Fe and VP500-Fe were not observed in the FL matrix, which had a substantially higher initial phosphate concentration than SL. Changes in pH during contact with the real wastewater matrices are presented in Table 6.

In FL, both BCs increased pH relative to the control, with values stabilizing at approximately 8.10–8.20 after 20–30 min. In contrast, in SL the pH in the presence of BCs was initially lower than that of the control and showed comparatively smaller changes during contact, particularly for VP500-Fe. Thus, the pH profiles during contact with the BCs differed between the FL and SL matrices.

## 3. Discussion

### 3.1. Physicochemical and Structural Properties of Biochars

The physicochemical and structural properties of the investigated BCs, as well as their phosphate sorption behavior, varied considerably depending on feedstock type, pyrolysis temperature, and modification strategy. In particular, the response to pyrolysis and modification differed between the two feedstocks. This is consistent with previous studies identifying feedstock composition as one of the major factors governing the elemental composition, mineral fraction, and surface properties of BCs. Meta-analyses have shown that BCs derived from agricultural residues often differ substantially from woody BCs in their mineral composition and surface chemistry, which may contribute to differences in nutrient sorption behavior [19,20,21].

During pyrolysis, decomposition and devolatilization of the organic matrix are accompanied by structural shrinkage and cracking, contributing to pore formation and the development of a porous carbonaceous structure [14,22]. Song et al. (2024) reported that increasing the pyrolysis temperature from 300 to 700 °C promotes progressive carbonization and relative enrichment of the mineral fraction, while specific surface area may increase as a result of volatile matter release and micropore development [23]. In the present study, the C content of unmodified BCs ranged from approximately 24% in T500 to more than 57% in VP400. For both feedstocks, increasing the pyrolysis temperature from 400 to 500 °C was accompanied by decreases in C and N contents, whereas concentrations of several mineral elements increased, particularly in the T-derived BCs. This pattern is consistent with a greater relative contribution of the inorganic fraction at the higher pyrolysis temperature. In contrast to the trend frequently reported in the literature, S_BET_ decreased with increasing pyrolysis temperature in the unmodified and DT-containing BCs, whereas Fe-modified materials showed a different response. Angın (2013) similarly reported a non-linear response of surface area to pyrolysis temperature, with S_BET_ initially increasing, reaching a maximum at 500 °C, and subsequently decreasing at higher temperatures [24]. These observations indicate that changes in S_BET_ with pyrolysis temperature are not necessarily monotonic and may depend on feedstock composition, temperature range, and process conditions.

The pronounced decrease in S_BET_ observed in the DT-containing composites should not be attributed solely to a functional modification by DT. The composites consisted of approximately 67% BC and 33% DT by mass. Therefore, part of the changes in their bulk composition inherently resulted from replacement of the carbonaceous fraction with the mineral component. This dilution effect was particularly evident for C, for which the ratios of C content in the BC-DT composites to that in the corresponding unmodified BCs ranged from 0.625 to 0.680, closely matching the expected BC mass fraction, with similar trends observed for N, K, and Mg. Jiang et al. (2023) similarly attributed decreases in the C and N contents of BC-DT composites partly to dilution by the mineral fraction [25]. In addition, interactions between DT and the BC matrix, including partial pore obstruction, surface coverage, or particle agglomeration, may have contributed to the decrease in N_2_-accessible surface area [26]. In the present study, DT incorporation was confirmed by the presence of characteristic Si-O-Si and Si-O-Al bands in the FT-IR spectra. Therefore, the effects of DT incorporation should be interpreted as a combination of compositional dilution and possible structural modification.

The FT-IR spectra reflected both functional groups inherited from the lignocellulosic feedstocks and their progressive transformation during pyrolysis [27,28]. Bands in the 3000–2800 cm^−1^ region, assigned to aliphatic C-H stretching vibrations, were associated with aliphatic components of the organic matrix [14]. The weakening of the broad O-H band at approximately 3300 cm^−1^ and the aliphatic C-H bands around 2900 cm^−1^ after pyrolysis was consistent with dehydration, dehydroxylation, loss of volatile aliphatic constituents, and progressive carbonization [29]. A similar trend was reported by Zhang et al. (2023), who observed progressive attenuation of O-H and aliphatic C-H bands, together with a reduction in C-O and C-O-C spectral features, in BCs produced from four different feedstocks over a pyrolysis temperature range of 250–650 °C [30]. The 1800–1000 cm^−1^ region contained overlapping contributions from organic and mineral components, including carbonyl-, carboxyl-, and aromatic-related vibrations [27]. In particular, bands in the approximately 1600–1500 cm^−1^ region were associated with aromatic skeletal vibrations and were consistent with the presence of aromatic structures whose relative contribution may increase during pyrolysis [15].

### 3.2. Phosphate Sorption

The results indicate that phosphate sorption by the investigated BCs was more strongly associated with feedstock type than with pyrolysis temperature or modification strategy. The kinetic profiles showed the greatest increase in phosphate uptake during the first 40 min of contact, followed by a progressive decrease in sorption rate, with the systems showing relatively small subsequent changes toward the end of the 120 min experiment. Such behavior is consistent with a gradual reduction in the driving force for mass transfer and the progressive occupation of readily accessible sorption sites [31,32]. The equilibrium isotherms also exhibited clear feedstock-dependent differences. The most pronounced divergence in isotherm behavior was observed between the T-DT and VP-DT materials, indicating a strongly feedstock-dependent response to DT incorporation. The generally satisfactory fit of the Freundlich model for most variants was consistent with sorption on energetically heterogeneous sites [33].

Phosphate sorption capacities reported for BCs span a broad range and depend strongly on feedstock composition, modification strategy, and experimental conditions. A particularly relevant comparison is provided by Yao et al. (2013), who investigated engineered BC prepared from Mg-enriched tomato tissues and reported a maximum phosphate sorption capacity exceeding 100 mg·g^−1^ [34]. Their results showed that the high phosphate sorption performance was closely associated with Mg-containing phases in the material. This finding provides a relevant mechanistic reference for tomato-derived BCs. Wang et al. (2021) compared *Phragmites australis*-derived BCs loaded with Al, Ca, Fe, lanthanum (La), and Mg oxides and demonstrated that the incorporated metal strongly affected both phosphate sorption capacity and sorption kinetics [35]. Although Al-BC exhibited the highest maximum sorption capacity (219.87 mg·g^−1^), the authors recommended Mg-BC when both sorption capacity and adsorption rate were considered. In the present study, Mg and Ca were not deliberately introduced during modification. However, their higher endogenous concentrations in T-derived BCs may have contributed to the greater phosphate uptake observed for this feedstock series.

Studies of Fe-modified BCs further indicate that total Fe content alone is insufficient to predict phosphate sorption performance because the reactivity of Fe-containing materials depends on the mineral phase, surface accessibility, and physicochemical environment [36]. Zhang et al. (2020), for example, directly compared BC composites containing magnetite, ferrihydrite, goethite, and hematite and reported phosphate sorption capacities in the order BC-goethite > BC-ferrihydrite > BC-hematite > BC-magnetite [37]. This phase-dependent behavior is compatible with the absence of a clear monotonic relationship between total Fe content and *q_e_* in the present study. Similarly, the lack of a direct correspondence between S_BET_ and *q_e_* indicates that N_2_-accessible surface area alone was not sufficient to explain phosphate sorption.

The effect of DT incorporation was particularly feedstock-dependent. Despite the marked decrease in S_BET_ in the DT-containing composites, T-DT materials retained comparatively high phosphate sorption, whereas the corresponding VP-DT materials showed substantially weaker uptake. Previous studies have demonstrated the potential of DT as a mineral support or component of composite phosphate sorbents. However, its performance depends strongly on the presence and nature of additional active phases. Wu et al. (2023), for example, immobilized La-modified diatomite in sodium alginate hydrogel beads and obtained a maximum phosphate sorption capacity of 58.9 mg·g^−1^, with phosphate removal exceeding 92.5% in real wastewater tests [38]. Zhang et al. (2019) investigated a thermally modified zeolite-DT composite for nutrient removal from agricultural wastewater, further demonstrating the potential of DT-containing composite sorbents [39]. These systems, however, differ substantially from the mechanically prepared BC-DT composites investigated here and therefore provide only indirect comparisons. The contrasting behavior of T-DT and VP-DT may reflect differences in the mineral composition and interfacial chemistry of the respective BC-DT composites. Nevertheless, the available data do not allow the effects of simple mass dilution to be quantitatively separated from changes in sorption properties associated with BC-DT composite formation. Further studies should therefore compare DT incorporation before and after pyrolysis and systematically evaluate the effects of DT loading and pretreatment, together with more detailed characterization of the mineral and surface phases involved in phosphate retention.

Overall, the results indicate that phosphate sorption was associated with both structural properties and the mineral composition of the investigated BCs. Phosphate retention by BC-based sorbents has been attributed to several mechanisms, including ligand exchange, surface complexation, and precipitation of metal phosphate phases [1,40]. Ca- and Mg-containing phases may be particularly important because these cations can promote phosphate retention through surface interactions and the formation of sparingly soluble phosphate phases [41,42]. The positive correlations between *q_e_* and Ca and Mg contents observed in the present study therefore support a possible contribution of the mineral fraction to phosphate retention. In Fe-modified BCs, the substantial increase in S_BET_ and pore volume may have enhanced the accessibility of sorption sites. Taken together, these results suggest that phosphate sorption depended on the combined effects of feedstock-dependent mineral composition, pore structure, and surface chemistry.

### 3.3. Phosphate Removal from Real Wastewater Matrices

The behavior of the magnetic BCs in real wastewater matrices differed from that observed in the model solution. The contrasting phosphate removal profiles obtained for FL and SL indicate a matrix-dependent response. In real wastewater, phosphate retention may be affected by dissolved organic matter, coexisting ions, and pH, which can alter the accessibility and reactivity of sorption sites and may either inhibit phosphate uptake through competitive interactions or promote phosphate retention through complexation and the formation of phosphate-containing mineral phases. In the present study, the increase in pH observed in FL is consistent with the contribution of alkaline mineral constituents of the BCs, whereas the different response in SL suggests a stronger influence of matrix composition. Pap et al. (2025) observed convergence of final pH toward near-neutral values after phosphate adsorption onto biosolids-derived biochar [43], whereas Gong et al. (2025) reported pH shifts after adsorption that depended on the initial solution pH and were associated with changes in surface protonation and phosphate speciation [44]. Sulfate concentrations were approximately 120 mg·L^−1^ in FL and 73 mg·L^−1^ in SL, confirming the presence of sulfate as a potentially competing inorganic anion in both matrices. Previous studies have shown that the effect of SO_4_^2-^ on phosphate sorption by modified BCs is material-dependent [45]: sulfate may moderately reduce phosphate uptake in Ca-rich BCs, whereas only minor effects have been reported for some Fe/Ca- [46] or La-modified materials [47]. The wastewater matrices also differed markedly in their buffering capacity. Total alkalinity was approximately 6-fold higher in FL (1205.83 mg CaCO_3_·L^−1^) than in SL (197.78 mg CaCO_3_·L^−1^). Therefore, the substantially higher alkalinity of FL indicates a markedly greater acid-neutralizing and buffering capacity. This difference may be relevant to phosphate removal because carbonate alkalinity can influence phosphate retention through competition for reactive sites, pH buffering, and competition with phosphate for Ca^2+^ involved in mineral precipitation [48]. In contrast, total hardness was higher in SL than in FL. The higher hardness of SL suggests a greater role of Ca- and Mg-related interactions in phosphate removal through processes such as cation bridging or precipitation [49]. These differences may indicate that FL and SL provided chemically distinct environments for phosphate removal, with potentially different contributions of competing ions, buffering effects, and divalent cations. Previous studies have demonstrated the applicability of modified BCs for phosphate removal from real wastewater matrices, including eutrophic waters, cattle-slurry wastewater, and industrial effluents [15,50,51,52]. These findings highlight the importance of validating BC-based sorbents in real wastewater matrices, as their performance under such conditions cannot be reliably predicted solely from experiments conducted in model solutions. Future studies should therefore include a more comprehensive characterization of wastewater matrices to distinguish their individual effects on phosphate removal.

## 4. Materials and Methods

All reagents were of analytical grade (Chemland, Stargard, Poland). Deionized water was used for the preparation of all aqueous solutions.

### 4.1. Feedstocks

Two types of waste biomass were used for BC production: tomato plant residues (T; *Solanum lycopersicum* L., cv. Kmicic) and shea nut shells (VP; *Vitellaria paradoxa*). Tomato plant residues, comprising leaves, stems, and peduncles, were collected after the harvest of mature tomato fruits in cooperation with the Department of Horticulture, West Pomeranian University of Technology in Szczecin (Poland). Shea nut shells were supplied by Sanwill (Szczecin, Poland). The biomass was air-dried, ground, and sieved to a particle size of <1 mm. The physicochemical characteristics of the feedstocks are provided in the Supplementary Materials (Table S1).

### 4.2. Biochar Production and Modification

BCs were produced by slow pyrolysis at 400 and 500 °C for 2 h in a muffle furnace (LSM-01, SNOL, Utena, Lithuania) at a heating rate of 10 °C min^−1^ under oxygen-limited conditions in covered porcelain crucibles. The resulting BCs were homogenized and stored in sealed containers until further analysis. Unmodified BCs produced at 400 and 500 °C were designated T400, T500, VP400, and VP500, respectively.

The BCs were subjected to two modification procedures: (i) mechanical homogenization with diatomite (DT) at a BC:DT mass ratio of 2:1 (approximately 66.7% BC and 33.3% DT by mass) and (ii) Fe-based magnetic modification by a co-precipitation method using Fe^3+^ and Fe^2+^ chloride salts at a Fe^3+^:Fe^2+^ molar ratio of 2:1, following the procedure described by Trinh et al. (2019) [53]. Modified BCs were designated according to the feedstock, pyrolysis temperature, and modification type as follows: T400-DT, T400-Fe, T500-DT, T500-Fe, VP400-DT, VP400-Fe, VP500-DT, and VP500-Fe.

### 4.3. Physicochemical and Structural Characterization of Biochars

Following acid digestion in a 3:1 mixture of nitric acid and perchloric acid, selected elements were quantified by atomic absorption spectrometry (AAS) using an air–acetylene flame (Solar S4, Thermo Fisher Scientific, Waltham, MA, USA). C, N, and S contents were determined using an elemental analyzer (FlashSmart, Thermo Fisher Scientific, USA).

Surface functional groups were characterized by Fourier-transform infrared spectroscopy (FT-IR) using a Nicolet iS5 (Thermo Fisher Scientific, USA) equipped with a diamond attenuated total reflectance (ATR) accessory. Spectra were recorded over the wavenumber range of 4000–400 cm^−1^ at a resolution of 1 cm^−1^ using 32 scans per spectrum.

The textural properties of the BCs were determined from N_2_ adsorption–desorption isotherms using an Autosorb iQ gas sorption analyzer (Quantachrome Instruments, Boynton Beach, FL, USA). The specific surface area (S_BET_) was calculated using the Brunauer–Emmett–Teller (BET) method. The DFT-derived specific surface area (S_DFT_) and cumulative pore volume (V_DFT_) were determined from the N_2_ sorption data using density functional theory (DFT).

### 4.4. Phosphate Sorption Experiments

#### 4.4.1. Phosphate Sorption Kinetics

Phosphate sorption kinetics were investigated for all 12 BC variants. In each experiment, 0.75 g of BC was suspended in 150 mL of a potassium dihydrogen phosphate (KH_2_PO_4_) model solution with an initial phosphate concentration of 200 mg P·L^−1^. The initial pH was adjusted to 7.0 using HCl or NaOH solutions, and the suspensions were agitated at 150 rpm. Solution samples were collected after 1, 5, 10, 20, 30, 40, 60, 90, and 120 min of contact. Before spectrophotometric analysis, the samples were separated from the sorbent using phosphate-free filter paper. The experimental data were fitted using the pseudo-second-order (PSO) kinetic model (Equation (1)), which provided the best description of the sorption kinetics among the models tested, including the pseudo-first-order and intraparticle diffusion models:

(1) $$q_{t}=\frac{k_{2}\cdot q_{e}^{2}\cdot t}{1+k_{2}\cdot q_{e}\cdot t}$$

where *q_t_*—amount of phosphate sorbed per unit mass of sorbent at time *t* (mg·g^−1^), *k*_2_—pseudo-second-order rate constant (g·mg^−1^·min^−1^), *q_e_*—model-estimated sorption capacity predicted by the model (mg·g^−1^), *t*—contact time (min).

Model parameters were not interpreted when the experimental profiles did not allow reliable estimation of the equilibrium sorption capacity (*q_e_*) or when no tendency toward sorption equilibrium was observed within the investigated contact time. Representative kinetic profiles are presented for two selected BC variants, T500-Fe and VP500-Fe. These variants were subsequently evaluated in the real wastewater matrices. Results obtained for the remaining variants were used to evaluate their sorption performance and suitability for subsequent equilibrium experiments.

Phosphate concentration was determined spectrophotometrically using the ascorbic acid-molybdenum blue method at a wavelength of 700 nm with a UV–Vis spectrophotometer (Evolution 201, Thermo Fisher Scientific, USA). The amount of phosphate sorbed at time *t* (*q_t_*) was calculated according to Equation (2):

(2) $$q_{t}=\frac{(C_{0}-C_{t})\cdot V}{m}$$

where *C*_0_—initial phosphate concentration in solution (mg·L^−1^), *C_t_*—phosphate concentration in solution at time *t* (mg·L^−1^), *V*—solution volume (L), *m*—mass of biochar (g).

The model-predicted initial sorption rate was calculated according to Equation (3):

(3) $$h=k_{2}\cdot q_{e,calc}^{2}$$

where *h*—initial sorption rate predicted by the PSO model (mg∙g^−1^∙min^−1^), *q_e,calc_*—model-estimated sorption capacity (mg∙g^−1^).

#### 4.4.2. Phosphate Sorption Isotherms

Equilibrium phosphate sorption experiments were conducted at room temperature using a static batch system. BC variants were selected for equilibrium experiments based on the kinetic tests, specifically when the sorption profiles showed stabilization within the investigated contact time and allowed conditions approaching equilibrium to be reliably established. For each experiment, 0.1 g of BC was added to 40 mL of KH_2_PO_4_ model solution with initial phosphate concentrations of 10, 25, 50, 75, 100, 125, 150, 175, and 200 mg P·L^−1^. The initial pH was adjusted to 7.0 using HCl or NaOH, and the suspensions were agitated at 150 rpm for 2 h. After the contact period, the sorbent was separated from the solution using phosphate-free filter paper, and the equilibrium phosphate concentration in the filtrate was determined spectrophotometrically.

The equilibrium sorption data were fitted using the Langmuir (Equation (4)) and Freundlich (Equation (5)) isotherm models [33]:

(4) $$q_{e}=\frac{K_{L}{\cdot C}_{e}\cdot q_{max}}{1+K_{L}{\cdot C}_{e}}$$

where *K_L_*—Langmuir constant (L·mg^−1^), *C_e_*—equilibrium phosphate concentration in solution (mg·L^−1^), *q_max_*—Langmuir-estimated maximum sorption capacity (mg·g^−1^).

(5) $$q_{e}=K_{f}\cdot C_{e}^{\frac{1}{n}}$$

where *K_F_*—Freundlich sorption coefficient [(mg·g^−1^)(L·mg^−1^)^1/n^], 1/*n*—Freundlich heterogeneity parameter.

### 4.5. Phosphate Removal from Real Wastewater Matrices

Two real wastewater matrices were investigated: (i) feed-production leachate (FL) originating from a laying-hen feed production facility (Cargill, Wrocław, Poland) and (ii) sewage sludge leachate (SL) obtained from a mechanical–biological wastewater treatment plant (Przedsiębiorstwo Usług Wodnych i Sanitarnych, Nowogard, Poland). Both matrices were vacuum-filtered prior to the experiments to remove macroscopic suspended matter. Sulfate concentration was determined spectrophotometrically using a BaCl_2_-based turbidimetric method at 420 nm with a UV–Vis spectrophotometer (Evolution 201, Thermo Fisher Scientific, USA). Total alkalinity was determined by potentiometric titration with 0.05 M HCl, whereas total hardness was determined by titration with 0.01 M EDTA.

Phosphate removal experiments in the real wastewater matrices were conducted using two selected magnetic BCs, T500-Fe and VP500-Fe. These materials were selected based on their phosphate sorption performance in the model solution, stable kinetic profiles, high S_BET_ values, and representation of both feedstocks. For each experiment, 0.1 g of BC was added to 40 mL of the corresponding wastewater matrix, and the suspensions were agitated at 150 rpm. Solution samples were collected after 1, 5, 10, 20, 30, 60, and 90 min of contact. The pH of the wastewater matrices was monitored during the contact experiments at selected sampling times. Before analysis, the sorbent was separated using phosphate-free filter paper. Phosphate concentration was determined spectrophotometrically using the same colorimetric procedure as described for the model-solution experiments. Control samples containing the corresponding wastewater matrix without BC addition were analyzed in parallel.

### 4.6. Statistical Analysis

Results from analyses conducted in triplicate are presented as mean ± standard deviation. Sorption experiments were performed once (*n* = 1), whereas physicochemical and structural analyses were conducted in triplicate (*n* = 3). Differences among BC variants were evaluated using one-way analysis of variance (ANOVA). Homogeneity of variances was assessed using Levene’s test, and Tukey’s honestly significant difference (HSD) post hoc test was applied when the homogeneity assumption was satisfied. Differences were considered statistically significant at *p* ≤ 0.05.

The multivariate structure of the dataset was explored using principal component analysis (PCA). Prior to PCA, variables were standardized using z-scores. Associations among selected parameters were assessed using Spearman’s rank correlation coefficients (*r_s_*), and the resulting correlation matrix was visualized as a heatmap. The correlation analysis involving phosphate sorption was based on 8 BC variants (*n* = 8).

In addition, an exploratory decomposition of variability was performed to estimate the relative contributions of feedstock type, pyrolysis temperature, and modification type to (i) the overall variation in the investigated physicochemical and structural properties of the BCs and (ii) phosphate sorption capacity (*q_e_*). Prior to this analysis, the physicochemical and structural variables were standardized using z-scores. For the physicochemical and structural dataset (*n* = 12 BC variants), a separate model was fitted for each response variable, and the sums of squares (SS) attributable to the main effects were subsequently aggregated across all variables and expressed as proportions of the total aggregated corrected SS. Phosphate sorption capacity was analyzed separately using the same factorial framework for the 8 BC variants included in the sorption analysis (*n* = 8), with the contribution of each factor expressed as a proportion of the corrected total SS. The remaining SS represented variation not assigned to the three main effects, including interaction effects and residual variation. Owing to the exploratory nature of these analyses, the resulting percentages were treated as descriptive measures of variability. All statistical analyses were performed using OriginPro 2021b (OriginLab Corporation, Northampton, MA, USA).

## 5. Conclusions

The investigated BCs derived from T and VP differed substantially in their physicochemical and structural properties and phosphate sorption performance. Exploratory decomposition of variability indicated that the relative importance of the investigated factors differed between material properties and sorption behavior. For BC physicochemical and structural properties (*n* = 12), modification type represented the largest proportion of variability, whereas for phosphate sorption (*n* = 8), feedstock type represented the largest proportion of the observed variation. The isotherm results further revealed contrasting sorption patterns between the two feedstocks: T-DT materials exhibited comparatively high phosphate sorption, whereas Fe-modified materials performed better within the VP series. No single physicochemical parameter consistently explained phosphate uptake across the investigated BCs, indicating that sorption performance was associated with the combined effects of structural and chemical properties, particularly pore characteristics and mineral composition. Evaluation of selected magnetic BCs in real wastewater matrices demonstrated their ability to reduce phosphate concentrations under chemically complex conditions and revealed a clear matrix-dependent response.

From an application perspective, sorbent selection should therefore consider the characteristics of the target wastewater rather than rely primarily on performance observed in model solutions. Feedstock type, modification strategy, and sorbent properties should be selected in relation to matrix composition, while characterization of competing ions, dissolved organic matter, and other relevant wastewater constituents should accompany future sorption studies.

Further optimization of DT-containing materials should distinguish the contribution of simple mass dilution from changes associated with BC-DT composite formation by systematically varying DT loading and comparing different routes of DT incorporation. Phase- and surface-sensitive characterization, particularly XRD and XPS, should also be used to characterize DT- and Fe-containing mineral phases and their association with the BC matrix.

For practical phosphorus recovery, future studies should additionally address sorbent regeneration, repeated sorption–desorption cycles, and continuous-flow performance. If phosphate-loaded BCs are intended for subsequent nutrient reuse, agronomic validation should include phosphorus release, seed-germination and phytotoxicity assays, plant-availability studies, and ultimately soil or pot experiments. Such an approach would shift BC development from maximizing sorption capacity under model conditions toward designing sorbents for specific wastewater matrices and defined end-use pathways.

## Figures and Tables

**Table 1:** Elemental composition and C/N ratios of unmodified and modified biochars.

Sample	C [%]	N [%]	S [%]	C/N Ratio	K [mg·g^−1^]	Na [mg·g^−1^]	Ca [mg·g^−1^]	Mg [mg·g^−1^]	Fe [mg·g^−1^]
T400	47.65 ^b^ ± 3.26	3.25 ^a^ ± 0.32	0.12 ^c^ ± 0.02	14.69 ^d^ ± 0.45	37.60 ^f^ ± 0.85	6.74 ^c^ ± 0.17	76.95 ^b^ ± 4.16	23.07 ^b^ ± 0.54	0.63 ^c^ ± 0.04
T400-DT	31.86 ^cde^ ± 2.11	2.20 ^b^ ± 0.16	0.12 ^c^ ± 0.05	14.48 ^d^ ± 0.10	25.25 ^g^ ± 0.31	5.66 ^d^ ± 0.34	53.32 ^d^ ± 0.03	15.76 ^d^ ± 0.18	0.58 ^c^ ± 0.03
T400-Fe	22.60 ^fg^ ± 0.75	1.48 ^c^ ± 0.15	0.03 ^de^ ± 0.01	15.34 ^d^ ± 1.05	0.49 ^h^ ± 0.01	2.53 ^f^ ± 0.11	39.27 ^e^ ± 0.10	11.58 ^e^ ± 0.08	422.21 ^ab^ ± 8.73
T500	24.27 ^efg^ ± 1.96	1.15 ^c^ ± 0.14	0.07 ^cde^ ± 0.02	21.17 ^bc^ ± 0.88	97.21 ^a^ ± 0.64	16.00 ^a^ ± 0.08	202.90 ^a^ ± 0.56	59.36 ^a^ ± 0.58	1.05 ^c^ ± 0.05
T500-DT	16.51 ^g^ ± 0.98	0.45 ^d^ ± 0.08	0.21 ^ab^ ± 0.06	37.22 ^a^ ± 4.51	36.25 ^f^ ± 1.40	7.77 ^b^ ± 0.21	65.30 ^c^ ± 2.07	20.67 ^c^ ± 0.33	0.54 ^c^ ± 0.04
T500-Fe	19.01 ^g^ ± 1.27	0.50 ^d^ ± 0.04	0.06 ^cde^ ± 0.01	38.05 ^a^ ± 0.51	0.46 ^h^ ± 0.08	1.83 ^g^ ± 0.12	19.50 ^f^ ± 1.43	19.87 ^c^ ± 0.10	448.33 ^a^ ± 33.33
VP400	57.17 ^a^ ± 4.55	3.32 ^a^ ± 0.43	0.11 ^cd^ ± 0.01	17.30 ^cd^ ± 0.88	67.57 ^c^ ± 0.98	1.13 ^h^ ± 0.05	6.80 ^gh^ ± 0.51	6.80 ^g^ ± 0.05	1.99 ^c^ ± 0.05
VP400-DT	37.63 ^c^ ± 3.29	2.23 ^b^ ± 0.17	0.12 ^c^ ± 0.02	16.86 ^d^ ± 0.19	40.95 ^e^ ± 0.68	1.61 ^gh^ ± 0.10	5.098 ^h^ ± 0.001	4.52 ^h^ ± 0.09	2.12 ^c^ ± 0.01
VP400-Fe	27.16 ^def^ ± 2.01	1.54 ^c^ ± 0.16	0.00 ^e^ ± 0.00	17.67 ^cd^ ± 0.53	1.59 ^h^ ± 0.08	4.94 ^e^ ± 0.27	3.53 ^h^ ± 1.22	3.55 ^i^ ± 0.15	408.71 ^b^ ± 5.99
VP500	54.56 ^ab^ ± 4.23	2.42 ^b^ ± 0.21	0.23 ^a^ ± 0.05	22.56 ^b^ ± 0.21	92.00 ^b^ ± 3.24	1.27 ^h^ ± 0.17	10.55 ^g^ ± 0.33	9.34 ^f^ ± 0.22	4.06 ^c^ ± 0.35
VP500-DT	34.12 ^cd^ ± 2.57	1.53 ^c^ ± 0.18	0.14 ^bc^ ± 0.02	22.37 ^b^ ± 0.96	59.37 ^d^ ± 0.02	1.61 ^gh^ ± 0.05	5.43 ^h^ ± 0.04	6.10 ^g^ ± 0.02	2.20 ^c^ ± 0.38
VP500-Fe	27.92 ^def^ ± 1.88	1.24 ^c^ ± 0.10	0.00 ^e^ ± 0.00	22.53 ^b^ ± 0.30	1.14 ^h^ ± 0.06	2.49 ^f^ ± 0.04	3.66 ^h^ ± 1.34	3.77 ^hi^ ± 0.18	439.63 ^a^ ± 8.99

T—tomato plant residues; VP—shea nut shells. The suffixes -DT and -Fe denote DT-containing BC composites and Fe-modified BCs, respectively. C—carbon; N—nitrogen; S—sulfur; C/N—carbon-to-nitrogen ratio; K—potassium; Na—sodium; Ca—calcium; Mg—magnesium; Fe—iron. Different letters (a–i) within the same column indicate statistically significant differences according to Tukey’s HSD test at *p* ≤ 0.05.

**Table 2:** Specific surface areas and pore structure parameters of unmodified and modified biochars.

Sample	S_BET_ [m^2^·g^−1^]	V_DFT_ [cm^3^·g^−1^]	S_DFT_ [m^2^·g^−1^]
T400	${9.92\;}^{c}\;\pm$ 0.79	0.0285 ^d^ ± 0.0007	${6.92\;}^{c}\;\pm$ 0.43
T400-DT	${5.22\;}^{c}\;\pm$ 0.39	0.0180 ^d^ ± 0.0014	${4.12\;}^{c}\;\pm$ 0.17
T400-Fe	${127.4\;}^{a}\;\pm$ 22.36	${0.3320\;}^{a}\;\pm$ 0.0000	${97.79\;}^{a}\;\pm$ 14.74
T500	${6.85\;}^{c}\;\pm$ 0.14	0.0200 ^d^ ± 0.0000	${5.41\;}^{c}\;\pm$ 0.03
T500-DT	${4.29\;}^{c}\;\pm$ 0.86	0.0100 ^d^ ± 0.0028	${3.30\;}^{c}\;\pm$ 0.76
T500-Fe	${130.12\;}^{a}\;\pm$ 42.38	0.2370 ^b^ ± 0.0594	${107.61\;}^{a}\;\pm$ 19.39
VP400	${4.68\;}^{c}\;\pm$ 0.42	0.0085 ^d^ ± 0.0007	${2.70\;}^{c}\;\pm$ 0.00
VP400-DT	${1.40\;}^{c}\;\pm$ 0.54	0.0027 ^d^ ± 0.0015	${1.24\;}^{c}\;\pm$ 0.08
VP400-Fe	${51.71\;}^{\mathrm{bc}}\;\pm$ 3.33	${0.1510\;}^{c}\;\pm$ 0.0028	${45.28\;}^{b}\;\pm$ 0.17
VP500	${2.37\;}^{c}\;\pm$ 0.06	0.0075 ^d^ ± 0.0007	${1.86\;}^{c}\;\pm$ 0.08
VP500-DT	${1.21\;}^{c}\;\pm$ 0.29	${0.0030\;}^{d}\;\pm$ 0.0014	${1.09\;}^{c}\;\pm$ 0.43
VP500-Fe	${100.1\;}^{\mathrm{ab}}\;\pm$ 23.83	${0.1975\;}^{\mathrm{bc}}\;\pm$ 0.0092	${98.30\;}^{a}\;\pm$ 13.91

T—tomato plant residues; VP—shea nut shells. The suffixes -DT and -Fe denote DT-containing BC composites and Fe-modified BCs, respectively. S_BET_—BET specific surface area; V_DFT_—cumulative pore volume; S_DFT_—DFT surface area. Different letters (a–d) within the same column indicate statistically significant differences according to Tukey’s HSD test at *p* ≤ 0.05.

**Table 3:** Pseudo-second-order kinetic parameters for phosphate sorption onto the investigated biochars.

Sample		PSO			
	*q_e,exp_* [mg·g^−1^]	*q_e,calc_* [mg·g^−1^]	*k*_2_ [g·mg^−1^·min^−1^]	*h* [mg·g^−1^·min^−1^]	*R* ^2^
T400	n.d.				
T400-DT	9.56	10.32	0.0121	1.289	0.973
T400-Fe	11.31	11.51	0.0136	1.802	0.983
T500	n.d.				
T500-DT	15.75	16.98	0.0030	0.865	0.945
T500-Fe	11.92	12.56	0.0122	1.925	0.988
VP400	n.d.				
VP400-DT	2.62	2.63	0.1028	0.711	0.955
VP400-Fe	4.65	4.91	0.0903	2.177	0.988
VP500	n.d.				
VP500-DT	0.97	1.05	0.0766	0.084	0.908
VP500-Fe	4.28	6.24	0.0294	1.145	0.962

T—tomato plant residues; VP—shea nut shells. The suffixes -DT and -Fe denote DT-containing composites and Fe-modified BCs, respectively. *q_e,exp_*—experimentally determined sorption amount at 120 min; *q_e,calc_*—model-estimated equilibrium sorption capacity; *k*_2_—pseudo-second-order rate constant; *h*—initial sorption rate; *R*^2^—coefficient of determination; n.d.—not determined. Parameters were not reported when the PSO model provided an unsatisfactory or unstable fit to the experimental data.

**Table 4:** Langmuir and Freundlich isotherm parameters for phosphate sorption onto modified biochars.

Sample	Langmuir			Freundlich		
	*q_max_* [mg·g^−1^]	*K_L_* [L·mg^−1^]	*R* ^2^	1/*n*	*K_F_* [(mg·g^−1^)(L·mg^−1^)^1/n^]	*R* ^2^
T400-DT	23.23	0.0404	0.872	0.354	3.569	0.915
T400-Fe	15.90	0.0792	0.698	0.274	3.922	0.912
T500-DT	39.48	0.0809	0.983	0.271	10.36	0.960
T500-Fe	9.85	0.4640	0.586	0.114	6.202	0.640
VP400-DT	148.62	0.0003	0.702	1.000	0.042	0.986
VP400-Fe	13.18	0.0077	0.795	0.483	0.651	0.982
VP500-DT	185.42	0.0002	0.445	1.125	0.017	0.722
VP500-Fe	26.39	0.0042	0.800	0.659	0.392	0.924

T—tomato plant residues; VP—shea nut shells. The suffixes -DT and -Fe denote DT-containing composites and Fe-modified BCs, respectively. *q_max_*—Langmuir-estimated maximum sorption capacity; *K_L_*—Langmuir constant; 1/*n*—Freundlich heterogeneity factor; *K_F_*—Freundlich sorption coefficient; *R*^2^—coefficient of determination.

**Table 5:** Physicochemical characteristics of the real wastewater matrices.

Parameter	FL	SL
Color [PCU]	${271.67\;}^{a}\;\pm$ 2.08	${77.67\;}^{b}\;\pm$ 4.51
Turbidity [NTU]	${14.70\;}^{a}\;\pm$ 0.35	${1.73\;}^{b}\;\pm$ 0.16
pH	${7.01\;}^{b}\;\pm$ 0.01	${8.59\;}^{a}\;\pm$ 0.01
EC [mS·cm^−1^]	${4.75\;}^{a}\;\pm$ 0.09	${2.42\;}^{b}\;\pm$ 0.01
$P-{{\mathrm{PO}}_{4}}^{3-}\;[\mathrm{mg}\cdot$L^−1^]	${192.82\;}^{a}\;\pm$ 28.56	${35.21\;}^{b}\;\pm$ 2.51
${{\mathrm{SO}}_{4}}^{2-}\;[\mathrm{mg}\cdot$L^−1^]	${119.17\;}^{a}\;\pm$ 10.70	${73.14\;}^{b}\;\pm$ 4.46
Total alkalinity $[\mathrm{mg}\;{\mathrm{CaCO}}_{3}\cdot$L^−1^]	${1205.83\;}^{a}\;\pm$ 3.82	${197.78\;}^{b}\;\pm$ 2.41
Total hardness $[\mathrm{mg}\;{\mathrm{CaCO}}_{3}\cdot$L^−1^]	${61.72\;}^{b}\;\pm$ 1.44	${84.24\;}^{a}\;\pm$ 1.44

FL—feed-production leachate; SL—sewage sludge leachate; EC—electrical conductivity. Different letters (a–b) within the same row indicate statistically significant differences at *p* ≤ 0.05.

**Table 6:** Changes in pH during sorption experiments with real wastewater matrices.

*t* [Min]	FL			SL		
	T500-Fe	VP500-Fe	Control	T500-Fe	VP500-Fe	Control
1	7.83 ± 0.01	7.79 ± 0.01	7.34 ± 0.01	8.02 ± 0.01	7.80 ± 0.01	8.35 ± 0.01
5	8.12 ± 0.01	7.86 ± 0.01		7.97 ± 0.01	7.76 ± 0.01	
10	8.22 ± 0.02	7.90 ± 0.01		7.87 ± 0.04	7.75 ± 0.01	
20	8.10 ± 0.03	8.19 ± 0.01		8.14 ± 0.02	7.75 ± 0.02	
30	8.17 ± 0.02	8.20 ± 0.02	7.34 ± 0.01	8.05 ± 0.01	7.80 ± 0.01	8.38 ± 0.01
60	8.19 ± 0.01	8.22 ± 0.02		8.15 ± 0.02	7.73 ± 0.03	
90	8.19 ± 0.01	8.26 ± 0.02	7.23 ± 0.01	8.27 ± 0.01	7.70 ± 0.01	8.29 ± 0.01

T—tomato plant residues; VP—shea nut shells. The suffix -Fe denotes Fe-modified BCs. *t*—time; FL—feed-production leachate; SL—sludge leachate. Control pH was measured at 1, 30, and 90 min.

## Data Availability

The raw data supporting the conclusions of this article will be made available by the authors on request.

## Supplementary Materials

The following supporting information can be downloaded at https://www.mdpi.com/article/10.3390/molecules31183277/s1, Figure S1: FT-IR spectrum of diatomite (DT); Table S1: Physicochemical properties of the feedstocks; Table S2: Exploratory decomposition of variability in the physicochemical and structural properties of biochars according to feedstock, pyrolysis temperature, and modification; Table S3: Exploratory decomposition of variability in experimentally determined phosphate sorption capacity according to feedstock, pyrolysis temperature, modification, and interactions.
