# Supplementary material for: Functionalized Biochars Derived from Tomato Plant Residues (Solanum lycopersicum L.) and Shea Nut Shells (Vitellaria paradoxa) for Phosphorus Sorption: From Model Solutions to Real Wastewater Matrices
Source: Molecules. 2026 Sep 16;31(18):3277. doi: 10.3390/molecules31183277 (PMC13609412; doi:10.3390/molecules31183277)
Supplement: Supplementary file 1 [file molecules-31-03277-s001.zip › Supplementary materials.pdf]

Article

Functionalized Biochars Derived from Tomato Plant Residues (*Solanum lycopersicum* L.) and Shea Nut Shells (*Vitellaria paradoxa*) for Phosphorus Sorption: From Model Solutions to Real Wastewater Matrices

Maja Owczarek [1,*], Hanna Siwek [1] and Kacper Świechowski [2]

[1] Department of Bioengineering, West Pomeranian University of Technology in Szczecin, Słowackiego 17, 71-434 Szczecin, Poland; hanna.siwek@zut.edu.pl
[2] Department of Applied Bioeconomy, Wrocław University of Environmental and Life Sciences, Chełmońskiego 37a, 51-630 Wrocław, Poland; kacper.swiechowski@upwr.edu.pl
*Correspondence: maja.owczarek@zut.edu.pl

Supplementary Materials

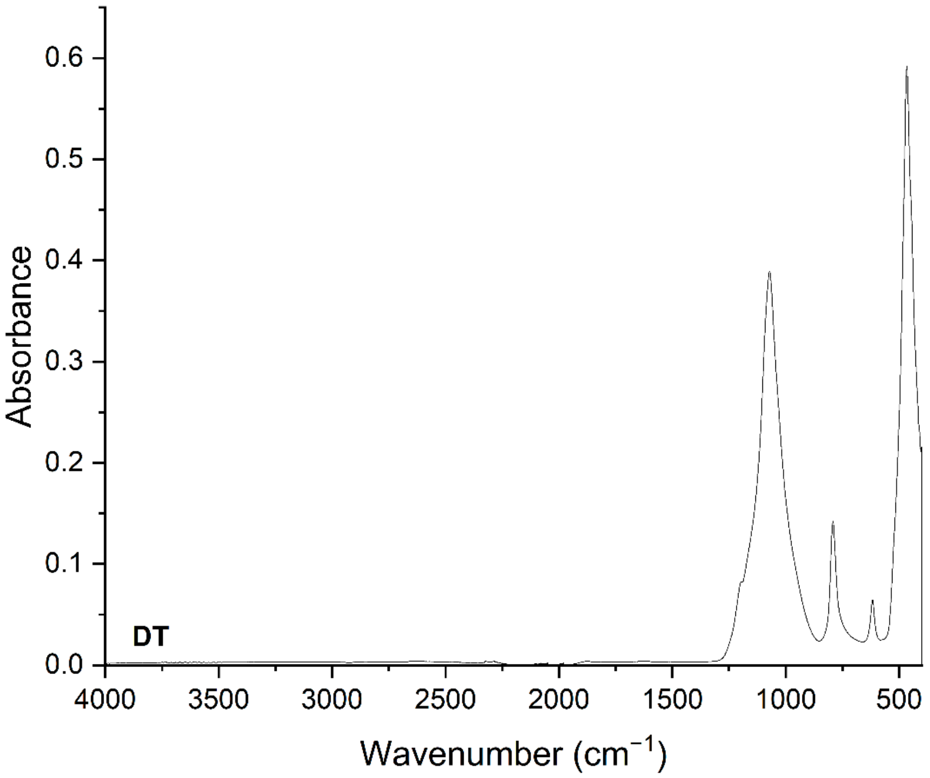

Figure S1. FT-IR spectrum of diatomite (DT).

Table S1. Physicochemical properties of the feedstocks.

Parameter	T	VP
DM [%]	90.44ᵃ ± 0.18	90.75ᵃ ± 0.27
Ash [%]	15.28ᵇ ± 0.03	7.76ᵃ ± 0.19
C [%]	39.95ᵃ ± 5.05	41.50ᵃ ± 0.64
N [%]	2.10ᵃ ± 0.22	2.44ᵃ ± 0.06
S [%]	1.00ᵇ ± 0.05	0.28ᵃ ± 0.04

C/N Ratio	18.97^{a} ± 0.40	17.03^{a} ± 0.07
P [mg·g^{-1}]	1.31^{a} ± 0.03	1.69^{b} ± 0.03
K [mg·g^{-1}]	11.24^{a} ± 0.38	23.57^{b} ± 0.04
Mg [mg·g^{-1}]	6.41^{b} ± 0.21	2.26^{a} ± 0.06
Ca [mg·g^{-1}]	19.01^{b} ± 0.25	0.49^{a} ± 0.03
Fe [mg·g^{-1}]	0.370^{a} ± 0.017	0.504^{b} ± 0.025
Pb [mg·g^{-1}]	0.0014^{a} ± 0.0002	0.0023^{a} ± 0.0002
Cd [mg·g^{-1}]	0.0014^{b} ± 0.0001	0.00050^{a} ± 0.00004

T—tomato plant residues; VP—shea nut shells. DM—dry matter; C—carbon; N—nitrogen; S—sulfur; C/N—carbon-to-nitrogen ratio; P—phosphorus; K—potassium; Mg—magnesium; Ca—calcium; Fe—iron; Pb—lead; Cd—cadmium. Different letters (a–b) within the same row indicate statistically significant differences according to Tukey's HSD test at $p \leq 0.05$.

Table S2. Exploratory decomposition of variability in the physicochemical and structural properties of biochars according to feedstock, pyrolysis temperature, and modification.

Variable	Feedstock SS	Temperature SS	Modification SS	Remaining SS
C	2.696	1.044	5.574	1.687
S	0.003	0.801	6.911	3.285
Ca	4.518	0.380	2.172	3.930
Mg	4.682	1.004	2.183	3.130
K	0.292	0.872	8.677	1.160
Fe	0.0005	0.006	10.979	0.014
S_{BET}	0.464	0.062	9.507	0.967
Sum of SS	12.655	4.169	46.003	14.173
Contribution [%]	16.42	5.41	59.75	18.41

SS—sum of squares. C—carbon; S—sulfur; Ca—calcium; Mg—magnesium; K—potassium; Fe—iron; S_{BET}—BET specific surface area. Remaining SS includes interaction effects and residual variation.

Table S3. Exploratory decomposition of variability in experimentally determined phosphate sorption capacity according to feedstock, pyrolysis temperature, modification, and interactions.

Source of variation	SS	Contribution [%]
Feedstock	162.18	84.84
Pyrolysis temperature	2.86	1.49
Modification	1.33	0.69
Feedstock × Pyrolysis temperature	9.72	5.09
Feedstock × Modification	6.88	3.60
Pyrolysis temperature × Modification	2.31	1.21
Feedstock × Pyrolysis temperature × Modification	5.88	3.08
Total	191.16	100.00

SS—sum of squares.
